# Study on dynamic changes of microbial community and lignocellulose transformation mechanism during green waste composting

**DOI:** 10.1002/elsc.202100102

**Published:** 2022-02-05

**Authors:** Yushan Zhang, Mengting Chen, Jingyi Guo, Ning Liu, Weiyi Yi, Zhongtai Yuan, Lifan Zeng

**Affiliations:** ^1^ College of Materials and Food Zhongshan Institute, University of Electronic Science and Technology of China Zhongshan P. R. China

**Keywords:** composting, green waste, lignocellulose, representative genera

## Abstract

There are few reports on the material transformation and dominant microorganisms in the process of greening waste (GW) composting. In this study, the target microbial community succession and material transformation were studied in GW composting by using MiSeq sequencing and PICRUSt tools. The results showed that the composting process could be divided into four phases. Each phase of the composting appeared in turn and was unable to jump. In the calefactive phase, microorganisms decompose small molecular organics such as FA to accelerate the arrival of the thermophilic phase. In the thermophilic phase, thermophilic microorganisms decompose HA and lignocellulose to produce FA. While in the cooling phase, microorganisms degrade HA and FA for growth and reproduction. In the maturation phase, microorganisms synthesize humus using FA, amino acid and lignin nuclei as precursors. In the four phases of the composting, different representative genera of bacteria and fungi were detected. S*treptomyces*, *Myceliophthora* and *Aspergillus*, maintained high abundance in all phases of the compost. Correlation analysis indicated that bacteria, actinomycetes and fungi had synergistic effect on the degradation of lignocellulose. Therefore, it can accelerate the compost process by maintaining the thermophilic phase and adding a certain amount of FA in the maturation phase.

**Abbreviations**
C/N, the total organic carbon/total nitrogen; EC, electrical conductivity
FA: fulvic acid
GW: green waste
HA: humic acid
HA/FA: humic acid/fulvic acid
TN: total nitrogen
TOC: the total organic carbon

## 1 INTRODUCTION

With the rapid development of urbanization and urban greening, large quantities of green waste (GW) have been produced. Urban GW mainly includes dead branches, pruning branches, fallen leaves, grass debris, falling flowers, etc., which occupy a large area in the expensive urban districts [1]. So far, there are still incineration and landfill to deal with GW in rural China since there is no effective treatment method. However, both of them not only pollute the environment, but also dissipate the bio‐resources. Plant biomass of GW usually consists of various polymers of cellulose (45%–55%), hemicellulose (25%–35%), and lignin (20%–30%) [2]. The efficient utilization of plant biomass will play a basic role in a future bio‐economy that relies on renewable carbon sources [3].

Composting is an economic and effective efficient way for utilization of GW. It is a biochemical as well as heterogeneous process involving mineralization of organic matter to CO_2_, NH_3_, H_2_O, and incomplete humification resulting in a stabilized final product with reduced toxicity and pathogenic organisms [4]. The environmental factors (temperature, moisture, aeration rate, pH, C/N ratio, etc.) dramatically change during composting process, which could influence microbial community and metabolism activity [5]. Temperature is a significant factor in determining the relative advantage of some microbial population over another. Previous researcher divided composting process into four phases namely mesophilic, thermophilic, cooling, and maturation stage according to the temperature evolution [6]. This is corresponding to the mineralization stage and humification stage of GW composting.

There have been a lot of reports on agricultural waste composting and microbial communities involved, such as the maize straw composting process [7], the wheat straw composting process [8], the rice straw process [9] corn stalk composting [10]. It was reported that *Firmicutes*, *Proteobacteria*, *Bacteroidetes*, *Chloroflexi*, *Actinobacteria*, and *Planctomycetes* were the dominant phyla during co‐composting of cow manure and maize straw [5, 11]. However, the microbial diversity during composting may vary with the variety of composting materials and nutrient supplements [12].  Therefore, it is necessary to study the diversity of microorganisms during GW composting. Phylogenetic investigation of communities by reconstruction of unobserved states (PICRUSt) was developed and has been widely used to determine microbial function succession in composting process [5, 7, 9].

**PRACTICAL APPLCATION**

Accelerating the degradation of lignocellulose can shorten the composting process of greening waste (GW). This research studied the composting process of GW from the perspective of microbial community succession and material transformation. The results show that bacteria, actinomycetes, and fungi had synergistic effect on the degradation of lignocellulose. In the stage of mineralization and humification, FA and HA are transformed into each other. In the thermophilic phase, the thermophilic microorganisms degrade HA to synthesize FA, while in the humification stage, FA is involved to synthesize HA. This indicates that FA produced during mineralization stage provides energy material for HA synthesis and decomposition of lignocellulose during humification stage. Therefore, providing a certain amount of FA in the humification stage can speed up the composting maturity process.

Composting takes a lot of time due to the large amount of lignocellulose in GW. Therefore, accelerating the conversion of lignocellulose to humus is the key to the full maturity of compost. The degradation of lignocellulose in GW composting process needs the joint action of microorganisms. Yet representative microorganisms in mineralization and humification stages during GW composting are still poorly understood. The research on lignocellulose transformation in the GW composting process is not deep enough.

Hence, the main objectives of this study were (i) to detect representative genera of microorganisms in the phases of mineralization and humification during GW composting, (ii) to study transforming mechanism of lignocellulose during the GW composting, (iii) to reveal the function of each phase of GW composting.

## 2 MATERIALS AND METHODS

### 2.1 Composting materials and experimental methods

The green waste was collected from a local city (Zhongshan, China). The green waste consist of 1/4 volume fresh and dead leaves, and 1/4 volume grass clippings, 1/4 volume fresh branches and 1/4 volume dead branches. The main characteristics of the initial materials are presented in Table 1.

**TABLE 1:** The physical‐chemical characteristics of the initial materials

Parameters	Green waste
pH	4.52 ± 0.31
EC (μS cm^–1^)	1540 ± 8.43
TN (g kg^–1^)	13.4 ± 0.64
Humus (%)	4.67 ± 0.78
HA (%)	1.57 ± 0.27
FA (%)	3.12 ± 0.69
TOC (g kg^–1^)	537.47 ± 7.48
C/N	40.11 ± 1.54
Cellulose (%)	44.24 ± 0.87
Hemicellulose (%)	23.47 ± 0.48
Lignin (%)	27.32 ± 0.32

Green waste was shredded into particles of 1–2 mm diameter by using wood shredder and straw shredder. The composting piles were placed in cuboid like‐shaped pile (3 m long, 2 m wide, and 1.4 m high), which were manually turned on days 6 and 12. The initial C/N ratio of the composting material was adjusted to 30:1by adding urea [7]. Moisture was maintained at 65% throughout the composting process [7]. A total 5% of wood biochar (w/w) was added to the compost material [9]. The composting was aerated using turning periodically. Digital thermometers were inserted in the mid of the composting piles to record temperature periodically. Composting samples were collected on days 1, 2, 3, 5, 12, 15, 18, and 21 and were labeled as S1, S2, S3, S5, S12, S15, S18, and S21. Samples were taken on the middle three cross sections of the area between 30 cm from the bottom and 30 cm from the top (the temperature change in this area was stable), then the samples were mixed evenly. The samples were divided into two parts: one part was stored at 4°C; the other was stored at –80°C for further analysis.

### 2.2 Physico‐chemical parameter analysis

The temperature of composting pile and surrounding environment were recorded every day. The pH and electrical conductivity (EC) were measured in the water extract from the sample according to the procedures described by previous researchers [13]. The contents of total nitrogen (TN) after drying, crushing, and sieving, the samples were digested with H_2_SO_4_‐H_2_O_2_ [14]. The total organic carbon (TOC) in the sample was determined by oil bath and potassium dichromate‐sulfuric acid oxidation method. Humus, humic acid (HA), and fulvic acid (FA) were determined according to previous researchers [15]. Subsequently humic acid/fulvic acid (HA/FA) was obtained base on HA and FA data. All samples were repeated three times.

### 2.3 DNA extraction and high‐throughput sequencing

DNA was extracted from the compost samples with the Powersoil DNA Isolation Kit (MO‐BIO Laboratories, Carlsbad, CA, USA) according to the manufacturer's instructions.

High‐throughput sequencing was performed on an Illumina Miseq platform (PE300) at Shanghai Personal Biotechnology Co., Ltd, China, using the primers 338F and 806R for the bacterial V3‐V4 hyper variable region of the 16S rRNA gene (338F, 5′‐ACTCCTACGGGAGGCAGCA‐3′, 806R, 5′‐GGACTACHVGGGTWTCTAAT‐3′). For fungi, polymerase chain reactions (PCR) was used to amplify its internal transcribed spacer (ITS) region of ribosomal DNA followed previous researchers [16]. One percent agarose electrophoresis was used to verify the quality of DNA extracted and PCR products amplified. PCR reactions were conducted in final volume of 20 μL containing 0.2 μL of Taq DNA polymerase (5 U μL^−1^), 0.3 μL of each forward and reverse primers (0.5 μmol L^−1^), 2 μL of template DNA, 2 μL of buffer, 1.6 μL of dNTPs and ddH_2_O to the final volume. The PCR temperature program was initiated denaturation with 3 min at 94°C, followed by 30 cycles of 30 s at 94°C, 40 s at 55°C, 60 s at 72°C, and final elongation with 5 min at 72°C. The quality‐filtered sequences were clustered into operational taxonomic units (OTUs) by 97% thresholds [17].

### 2.4 Statistical analysis

All samples used for analyses were performed in triplicates. PICRUSt was used to explore the functional composition of that bacterial community data may convey. The nonparametric Spearman correlation coefficient was greater than 0.68 (*p* < 0.01). All the analyses were performed with IBM SPSS Statistics 23 for windows (IBM Corp., NY, USA). A *p*‐value < 0.05 was considered statistically significant.

## 3 RESULTS AND DISCUSSION

### 3.1 Changes in physicochemical characteristics during composting

**FIGURE 1:**
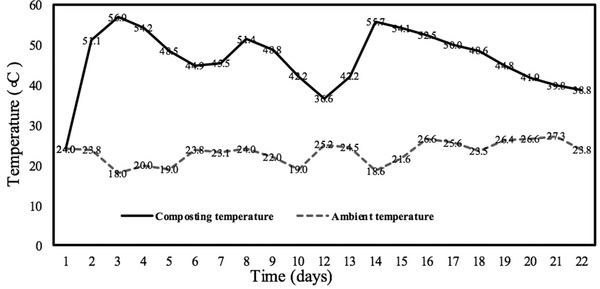
Temperature curve and division of four phases in GW composting process

Temperature is an important indicator of the composting process and plays a selective role in the succession of microbial communities [9]. In this study, four times of turning were carried out on days 6 and 12, respectively. Due to the more uniform mixing of materials and sufficient oxygen supply, the temperature of the composting pile fluctuated and form multiple circles (Figure 1). According to the temperature division of 40℃ in the thermophilic phase [18] and Cluster analysis results (data not shown), the whole composting process can be divided into four phases. As shown in Figure 1, the calefactive phase (day 1 to 2), the thermophilic phase (day 2 to 12), the calefactive phase (day 12 to 15), the cooling phase (day 15 to 18), and the maturation phase (day 18 to 21). The thermophilic phase (day 2 to 12) can be divided into two phases: the prophase of the thermophilic phase (day 2 to 6) and the anaphase of the thermophilic phase (day 6 to 12). Obviously, four phases, the calefactive phase, the thermophilic phase and the cooling phase, belong to the mineralization stage of the composting, while the maturation phase belongs to the humification stage. Therefore, taking 18 days as the boundary, the whole composting process was divided into mineralization stage (1–18 days) and humification stage (after day 18).

Because of the rapid decomposition of degradable organic matter by the action of microorganisms and the release of a large amount of heat, the calefactive phase is resulted in a fast increase in pile temperature [7]. After turning, the composting temperature affected the composting process.

**FIGURE 2:**
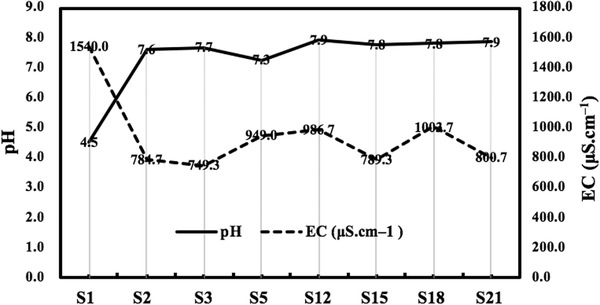
Changes of pH and EC value during GW composting

The changes in pH during the composting are shown in Figure 2. The pH in the initial material was 4.5 due to microorganisms’ production of organic acids from the decomposition of organic matter. During the GW composting, the pH value increased rapidly in the calefactive phase, then the pH value slightly decreased in the prophase of the thermophilic phase (day 2 to 5), then increased slightly in the anaphase of the thermophilic phase (day 5 to 12). Then the pH value gradually increased to 7.9. The decrease of pH value in the prophase of the thermophilic phase indicates that the release of inorganic salts comes from lignocellulose decomposed by thermophilic microorganism. The increase in the pH value may be induced by the release of a large amount of NH_4_^+^‐N from the decomposition of organic matter, such as protein‐ and nitrogen‐containing bases, by bacteria during the early composting process [19]. This indicates that the release of NH_4_^+^‐N and ammonia mainly occurs in the calefactive phase and the thermophilic phase of composting,. The pH value gradually stabilized in alkali values due to the volatilization of ammonia, the H^+^‐released from microbial nitrification, the decomposition of organic matter and production of organic and inorganic acids, and the release of carbon dioxide during the humification stage [20].

The EC reflects the total salt concentration of the compost sample and suggests whether phytotoxicity/plant inhibitory effects may be present in a sample [21]. As shown in Figure 2, the EC Value of the compost decreased rapidly in the calefactive phase. The decrease of EC value was also related to the rapid increase of pH value. This also implied that a large amount of NH_3_ was released during the calefactive phase. In the thermophilic phase, the EC value increased rapidly from 784.7 to 986.7 μS cm^–1^, and decreased in the calefactive phase (day 12 to 15). The EC value increased in the cooling phase, and gradually decreased to 800.7 μS cm^–1^ in the maturation phase. The rapid increase in EC from day 3 to 12 (the thermophilic phase) may be resulted from the release of mineral salts due to the degradation of organic matter.

The calefactive phase (day 12–15) was the phase of rapid microbial reproduction. At this time, new materials increase due to turning on the day 12 and the small molecular substances such as glucose produced in the thermophilic phase existed, the number of microorganisms increased rapidly and assimilated inorganic nitrogen into organic nitrogen, resulting in the decrease of EC value from 12 to 15 days. In the cooling phase (15–18 days), microorganisms decompose humic acid (HA and FA) and release mineral salts, resulting in the increase of EC value. The maturation phase (18–22 days) was the phase of humus synthesis. During this phase, microorganisms use lignin core, FA and amino acids as raw materials to synthesize macromolecular humus, resulting in the decrease of EC value.

The EC value of the day 22 compost (800.7 μS cm^–1^) was significantly lower than that of the initial phase (1200 μS cm^–1^). At the end of composting the EC value was lower, indicating that the composting quality was better, since an increase in EC would lead to phyto‐inhibitory effects in the soils and plants.

**FIGURE 3:**
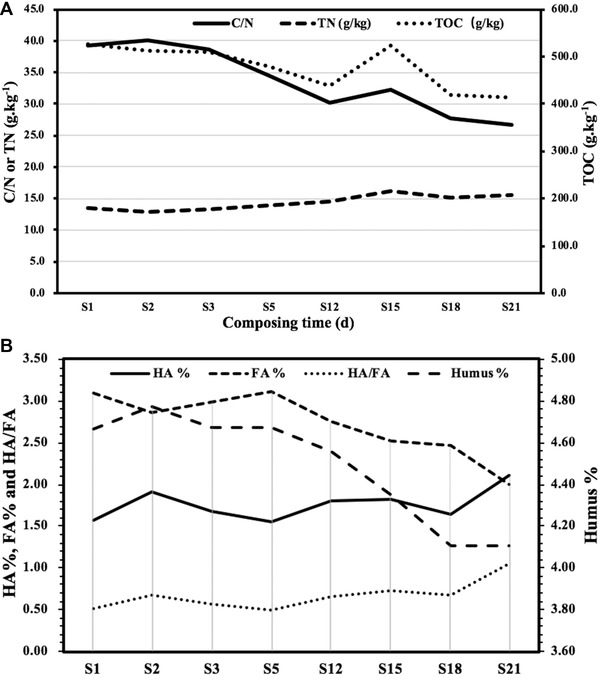
(A) Change of C/N, TN, and TOC content during composting of green waste. (B) Change of fulvic acid (FA), humic acid (HA), humus (%), and HA/FA ratio during the GW composting process

It can be seen from Figure 3A that TOC and C/N decreased rapidly in the first 12 days and gradually increased in the calefactive phase (day 12–15). Then TOC and C/N decreased gradually in the cooling phase (day 15–18) and gradually stabilized in the maturation phase. New materials were mixed into the composting pile due to the turning on day 12, which caused the TOC content and C/N increased rapidly from day 15 to 18. After 18 days the compost gradually entered the humification stage, a large number of nitrogen‐containing compounds such as protein and amino acid were used to synthesize humus [22], the increase of TN slowed down. This showed that organic matter is mainly degraded in the mineralization stage and the cooling phase.

TN content decreased slightly in the calefactive phase (day 0–2), increased rapidly from day 2 to 15, and gradually decreased in the cooling phase (day 15–18). TN the stabilized in the maturation phase (day 18–21). The content of TN decreased slowly during the calefactive phase (day 0–2) due to the release of ammonia and heat from degradable small molecules decomposition in the calefactive phase [22a]. The increase of TN from day 2 to 15 indicated continuous assimilation of nitrogen and decomposition of organic carbon.

In the presence of easily metabolized carbon sources, such as glucose, the expression of genes necessary for utilization of alternative carbon sources is repressed; it will prevent the microorganisms from producing β‐glucosidase [23, 24]. This is often called the effect of glucose inhibition. The growth and metabolism of microorganisms during the calefactive phase mainly depend on simple organic matter such as glucose. Cellulase and hemicellulase are produced very few in the calefactive phase due to the effect of glucose inhibition, resulting in the low degree of degradation of cellulose and hemicellulose in the calefactive phase. With the depletion of glucose, mesophiles were inhibited or even died. Thermophilic microorganisms became the dominant population when composting entered the thermophilic phase. During the thermophilic phase thermophilic microorganisms began to decompose lignocellulose and to multiply [25], which produced a large amount of CO_2_ and assimilated protein, resulting in the rapid decline of TOC content and the rapid rise of TN. Therefore, the thermophilic phase is the main phase of lignocellulose degradation. With the rapid propagation of thermophilic microorganisms, a large number of cellulases were produced, which means that microorganisms began to use macromolecular cellulose organic matter for catabolism.

As shown in Figure 3B, FA content decreased in the calefactive phase (day 1–2) while gradually increased in the prophase of the thermophilic phase (day 2–5), after day 5 FA content decreased rapidly.

However, HA content was just the opposite case. HA content increased in the calefactive phase (day 1–2) while gradually decreased in the prophase of the thermophilic phase (day 2–5) and increased in the anaphase of the thermophilic phase (day 5–12). HA content was decreased in the cooling phase (day 15–18) while sharply increased in the maturation phase (day 18–21). This indicated that FA was degraded like glucose and was used as an energy source by microorganisms and released CO_2_ during the calefactive phase, and the content of HA increased due to the concentration effect. With the increase of temperature, thermophilic microorganisms degraded macromolecular substances such as lignocellulose and HA and produced small molecular substances FA, which led to the increase of FA content and the decrease of HA content in the prophase of the thermophilic phase (day 2–5). The increase of HA content in the anaphase of the thermophilic phase (day 5–12) indicated that HA was synthesized by decomposing lignocellulose. It was reported that *Bacillus* was shown to play key roles in degrading high‐molecular‐weight organic substances into small‐molecular‐weight humic‐ and fulvic‐acid‐like substance [26]. This also implies that in the early stage of the thermophilic phase glucose and FA are obtained by decomposing HA in the material. With the accumulation of glucose, a large number of thermophilic microorganisms are produced. In the later stage of the thermophilic phase, thermophilic microorganisms begin to decompose lignocellulose to produce HA. This also implies that only a sufficient number must reach thermophilic microorganisms to degrade lignocellulose. In the cooling phase, mesophilic microorganisms mainly grow and reproduce by decomposing HA, resulting in the decrease of HA content. In the maturation phase, mesophilic microorganisms began to synthesize HA with the increase of the number of mesophilic microorganisms. This indicated that HA was mainly synthesized in the maturation phase and FA was involved in the synthesis of HA. FA with a small molecular weight and simple structure was used as an energy source by microorganisms, the FA was largely decomposed by microorganisms to produce HA [27].

The change of humus is also shown in Figure 3B. During the calefactive phase (day 0 to day 2), the humus content increased slightly, which may be due to the concentration effect after FA decomposition in this phase. The humus content decreased rapidly from day 2 to 18, and remained basically unchanged in the mature stage (18–21 days). Since HA and FA are the main components of humic acid, the change of humus was basically consistent with the changes of HA and FA in the process of composting. This shows that humus can be divided into mature humus and immature humus and that the humification of composting is the process of transforming immature humus into mature humus.

In this study, we especially emphasize that HA and FA plays a key role in the degradation of lignocellulose. Simple organics such as FA play a key role in maintaining the number of microorganisms and composting temperature in the initial phase and the calefactive phase; HA plays a key role in maintaining the number of microorganisms in the thermophilic phase and the cooling phase, so as to ensure that microorganisms decompose lignocellulose and synthesize humic acid. Carbohydrate metabolism during composting can produce various compounds through the degradation of hemicellulose and cellulose. When the C/N ratio decreased to a certain extent, the process of organic nitrogen oxidation was weakened, and the microbial organic nitrogen synthesis was enhanced [28]. With the decrease of temperature, humification increased and organic nitrogen was transformed into HA containing aromatic compounds [14]. A large number of studies have shown that amino acids can be used as raw materials for the synthesis of HA and directly promote the synthesis of humus [22, 29]. According to the polyphenol & protein theory, FA is first produced and is further condensed to produce HA [30]. It is suggested that both FA and amino acid are involved in the synthesis of HA. This can also explain why FA gradually decreased while HA and TN gradually increased in the humification stage. Generally speaking, the content of FA in immature compost is higher than that of HA, but the opposite is true in mature compost. HA was mainly produced in the maturation phase during the GW composting. Therefore, HA/FA ratio is a parameter used to evaluate the maturity of the final compost [31]. The change of HA/FA ratio was consistent with that of HA. The overall trend of HA/FA ratio increased gradually in the composting process, while increased rapidly from the 18th day. The change of HA/FA ratio was consistent with that of TN. In the humification stage, the formation of humus is mainly due to the combination of nitrogen‐containing compounds such as protein and amino acid and lignin degradation products [22].

In summary, the temperature, microbial genera, substrate and product are different in each phase of composting process. The substrates in the initial phase and in the calefactive phase are mainly simple organic substances such as glucose, FA, during which microorganisms degrade these simple substances to produce CO_2_, NH_3_, and heat. These two phases are period of activating and expanding the number of microorganisms. The substrate in the early stage of the thermophilic phase is mainly HA, and the substrate in the later stage of the thermophilic phase substrates is lignin, cellulose, hemicellulose. In the early stage of the thermophilic phase, HA is decomposed for the growth and reproduction of thermophilic microorganisms. In the later stage of the thermophilic phase, microorganisms begin to decompose lignocellulose to produce HA, FA, CO_2,_ lignin nuclei and heat, resulting in the continuous rise of temperature. When the decomposition of lignocellulose is completed and the temperature in the thermophilic phase exceeds 65℃, thermophilic microorganisms gradually sleep or die. Then the temperature gradually decreases and the compost enters the cooling phase. The substrates in the cooling phase are mainly HA and FA. In the cooling phase microorganisms decompose HA and FA produced in the thermophilic phase. As the number of microorganisms reaches a certain number in the cooling phase, microorganisms enter the maturation phase. The substrates in the maturation phase, such as lignin nuclei, amino acid and FA, are synthesize HA and humus.

### 3.2 Dynamics of bacterial community composition during composting

**FIGURE 4:**
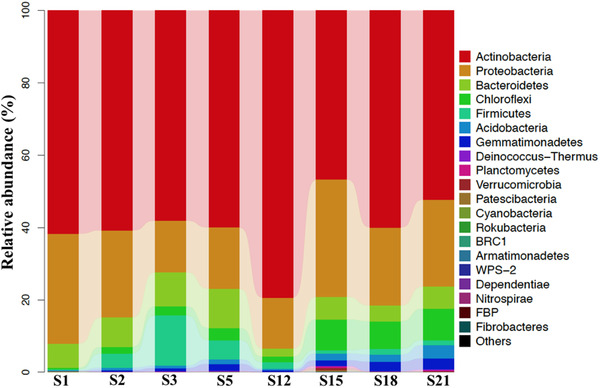
Dominant bacterial phyla detected in the process of **GW** composting

A total of 21 bacterial phyla were detected in the samples. Seven dominant phyla, Actinobacteria (59.9%), Proteobacteria (22.2%), Bacteroidetes (6.8%), Chloroflexi (4.3%), Firmicutes (3.6%), Acidobacteria (1.3%), and Gemmatimonadetes (1.3%), which accounted for 97.6% of the sequences, were the dominant phyla (Figure 4). The phylum Actinobacteria was the most abundant bacteria phylum identified and followed by Proteobacteria. The phyla, Actinobacteria, and Proteobacteria, were also found in tree decayed process [32]. Zhou et al. reported that Firmicutes*, *Actinobacteria and Proteobacteria were the dominant phyla in rice straw composing with pig manure [9]. This indicates that the types of composting materials affect the microbial community and relative abundances.

Firmicutes, Proteobacteria, Bacteroidetes, and Actinobacteria are considered to be the dominant phyla in lignocellulosic composting. These dominant taxonomic phyla were also found in maize straw composting process [7], in the wheat straw composting process [8] and in the rice straw composting process with pig manure [9], but the relative abundance of these phyla were different from that in this study. Compared with corn straw, wheat straw and rice straw, there is a higher content of cellulose in green waste.

**FIGURE 5:**
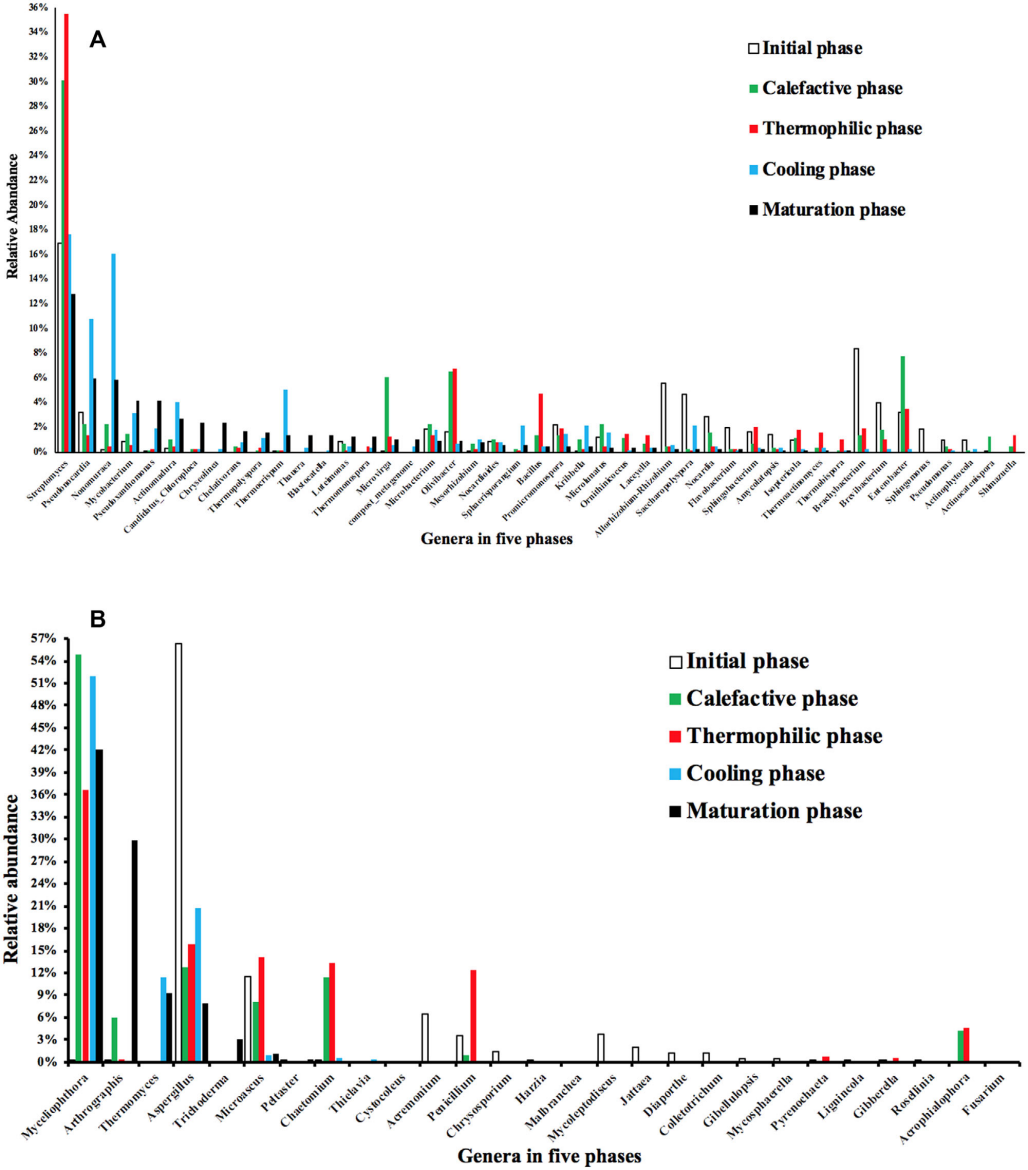
(A) Bacterial genera with abundance more than 1% were detected in the five composting phases. (B) Fungal genera with abundance more than 1% were detected in the five composting phases

At the genus level, total 45 bacterial genera with abundance more than 1.00% were detected in the calefactive phase, the thermophilic phase, the cooling phase and the maturation phase (Figure 5A). Thirty genera were detected in the initial phase, in which seven genera with abundance more than 3%, such as *Streptomyces*, *Brachybacterium*, *Allorhizobium‐Rhizobium*, *Saccharopolyspora*, *Brevibacterium*, *Pseudonocardia*, and *Enterobacter*, could be regarded as the representative genera of the initial phase. Thirty‐eight genera were detected in the calefactive phase, in which four genera with abundance more than 6%, such as *Streptomyces*, *Enterobacter*, *Olivibacter*, and *Microvirga*, could be regarded as the representative genera of the calefactive phase. Thirty‐eight genera were detected in the thermophilic phase, in which four genera with abundance more than 3%, such as *Streptomyces*, *Olivibacter*, *Bacillus*, and *Enterobacter*, could be regarded as the representative genera of the thermophilic phase. Forty‐two genera were detected in the cooling phase, in which six genera with abundance more than 3%, such as *Streptomyces*, *Nonomuraea*, *Pseudonocardia*, *Thermocrispum*, *Actinomadura*, and *Mycobacterium*, could be regarded as the representative genera of the cooling phase. Thirty‐seven genera were detected in the maturation phase, in which five genera with abundance more than 3%, such as *Streptomyces*, *Pseudonocardia*, *Nonomuraea*, *Mycobacterium*, and *Pseudoxanthomonas*, could be regarded as the representative genera of the maturation phase.

The genus from Actinobacteria, *Streptomyces*, had the highest abundance (32%) in all phases of the compost, especially has the highest abundance in the thermophilic phase. It was reported that two‐domain laccase‐like multicopper oxidase (LMCO) genes were detected in *Streptomyces spp*., which are potentially involved in bacterial extracellular phenol oxidase activities and lignocellulose breakdown during agricultural waste composting [33]. *Streptomyces spp* contributed to a higher temperature (maximum 66.8°C) and increased the TN, phosphorus, and potassium content in composts [34]. Previous reported that members of the genus *Olivibacter* are involved in the catechol catabolic pathway and the degradation of complex and toxic compounds and degrade phenolic compounds and hemicelluloses [35]. In this study, the genus *Enterobacter* only appeared in the mineralization stage (the initial phase, calefactive phase and thermophilic phase), indicating that the genus *Enterobacter* played an important role in composting mineralization. Xu et al. (2013) reported that *Enterobacter sp*. were the optimal fermentation strains for pig manure composting [36], which can prolong the thermophilic stage of composting, accelerate the decomposition rate of organic matter and accelerate the decline rate of C/N ratio. *Bacillus*, is often detected in lignocellulosic composting systems as thermotolerant bacteria [37], where it contributes to waste degradation during the composting process [7]. *Bacillus* was shown to play key roles in degrading high‐molecular‐weight organic substances into small‐molecular‐weight humic‐ and fulvic‐acid‐like substance [26].  It was reported that that *Bacillus* is related to lignin degradation based on genome and proteomic analysis [38]. This indicates that the genus *Bacillus* is an important genus that can degrade lignin during the mineralization stage. There are few reports about the genus *Microvirga*. In this study, this genus had a high abundance in the calefactive phase. Previous study reported the strains from *Microvirga* are alphaproteobacterial root‐nodule bacteria that specifically nodulate and fix nitrogen [39]. Therefore, the genus *Microvirga* may be related to the decomposition of organic matter and the reduction of C/N ratio during the mineralization stage.

Among the representative genera detected in the cooling phase, besides the genus *Streptomyces*, the top four genera were *Nonmuraea, Pseudonocardia, Pseudoxanthomonas*, and *Microbacterium*. Previous reported *Nonomuraea* species exhibited significantly positive relationships with extracellular enzyme activities such as cellulase and dehydrogenase, suggesting the *Nonomuraea* is involved in the degradation of cellulose and hemicellulose [40]. It was reported that the genus, *Pseudonocardia*, might play a notable role in the degradation of cellulose [41]. In this study, the abundance of *Pseudonocardia* was higher in the humification stage than that in the mineralization stage, indicating that this genus this genus played an important role in the formation of HA at maturity phase. The genus *Pseudoxanthomonas* has the ability to degrade all six BTEX (benzene, toluene, ethylbenzene, and o‐, m‐, and p‐xylene) compounds [42]. Lignin derives from various other aromatic monomers [43]. Lignin and its degradation products, such as phenolic compounds, quinone compounds, and aliphatic compounds, are the main precursors of humus formation during the humification stage, which form humus through complex reaction mechanisms [44]. This indicates that lignin degradation products are involved in the synthesis of humus on the one hand, and a part of BTEX compounds are degraded by *Pseudoxanthomonas* on the other hand, which may provide intermediate products of energy supply for other microorganisms in the humification stage. The genus *Microbacterium* was reported to be involved in lignocellulose degradation during lignocellulose‐based composting [45].

### 3.3 Dynamics of fungal community composition during composting

Two fungal phyla, Ascomycota and Basidiomycota, were detected in the composting process. The most abundant genus was Ascomycota, which could account for 99.4% of the total sequences. Ascomycota can secrete a variety of cellulose and hemicellulose degrading enzymes, which can efficiently utilize the nutrient elements in compost [46]. Ascomycota includes fungi with high lignin degradation activity, and the representative fungi were *Aspergillus* and *Penicillium* [47].

At the genus level, total 27 fungal genera with abundance more than 1.00% were detected the initial phase, the calefactive phase, the thermophilic phase, the cooling phase and the maturation phase (Figure 5B). Ten genera were detected in the initial phase, in which six genera with abundance more than 2%, such as *Aspergillus*, *Microascus*, *Acremonium*, *Mycoleptodiscus*, *Penicillium*, and *Jattaea*, could be regarded as the representative genera of the initial phase. Eleven genera were detected in the calefactive phase, in which six genera with abundance more than 4%, such as *Myceliophthora*, *Aspergillus*, *Chaetomium*, *Microascus*, *Arthrographis*, and *Acrophialophora*, could be regarded as the representative genera of the calefactive phase. Twelve genera were detected in the thermophilic phase, in which sic genera with abundance more than 4%, such as *Myceliophthora*, *Aspergillus*, *Microascus*, *Chaetomium*, *Penicillium*, and *Acrophialophora*, could be regarded as the representative genera of the thermophilic phase. Eleven genera were detected in the cooling phase, in which three genera with abundance more than 11%, such as *Myceliophthora*, *Aspergillus*, and *Thermomyces*, could be regarded as the representative genera of the cooling phase. Fifteen genera were detected in the maturation phase, in which five genera with abundance more than 3%, such as *Myceliophthora*, *Arthrographis*, *Thermomyce*, *Aspergillus*, and *Trichoderma*, could be regarded as the representative genera of the maturation phase.

Two fungal genera, *Myceliophthora* and *Aspergillus*, maintained high abundance at both mineralization and humification stages, indicating that the two genera are multifunctional. The 27 fungal genera were detected during the composting, which can be divided into three categories according to their niche. The first category is the genera with high abundance in both mineralization stage and humification stage, which is the dominant genus throughout the whole composting stage, such as *Myceliophthora* and *Aspergillus*. The second category is the genera with high abundance in the mineralization stage and low abundance in the humification stage, which are representative genera of the mineralization stage, such as *Chaetomium*, *Microascus*, *Penicillium*, and *Acrophialophora*. The third category is the genera with high abundance in the humification stage and low abundance in the mineralization stage, which are representative genera of the humification stage, such as *Arthrographis* and *Thermomyces*.

As the most abundant fungal genus, *Myceliophthora*, is predominantly involved in xylan degradation and xylose catabolism [48, 49] and can produce a set of cellulolytic enzymes to degrade cellulose synergistically [50, 51]. Xylan is the main component of plant hemicellulose. The gene sequence of the strain from *Myceliophthora* contains a large number of genes encoding lignocellulose decomposing enzymes, which can secrete a large number of thermostable carbohydrate decomposing enzymes and lignin decomposing enzymes, thus degrading various biomass [5, 49, 52]. This indicates that the genus *Myceliophthora* is the main genus that decomposes hemicellulose and lignin of green waste.

As the second most abundant genus, *Aspergillus* is considered as the core genus of lignocellulose degradation at the thermophilic phase during the co‐composting of rice straw and swine manure [53]. The genus *Aspergillus* can degrade lignin efficiently in GW composting [47]. It was reported that strains from *Chaetomium* can produce laccase during the thermophilic stage of composting, which can remain active for a long period of time at high temperatures and alkaline pH values and is involved in the humification process during composting [54]. This indicates that this genus *Chaetomium* is the one that degrades lignin in the mineralization stage. It was reported that *Penicillium subrubescens* produced a variable set of (hemi‐) cellulolytic activities on plant biomass substrates with activity levels comparable to those of *Aspergillus niger* [55]. During lignocellulose‐based composting, the genera *Penicillium* and *Microascus* were detected at all the composting phases and showed the highest relative abundances [56]. It was reported that the species of *Acrophialophora* can produce Xylanase and endo‐1‐4‐beta‐glucanase and play an essential role in the degradation of lignocellulose [57, 58].

As a representative genus of humification stage, *Arthrographis* showed an inverse correlation with indicators of organic matter stabilization in a full‐scale composting pile with sewage sludge and a vegetal bulking agent [59]. In this study, the relative abundance of the genus of *Arthrographis* in the maturation phase was very high (29.9%), suggesting that this genus may also play an important role in the formation of humus. The genus *Thermomyces* was also reported as the dominant genus that might be the main functional groups to degrade hemicellulose in mesophilic, thermophilic, and mature phase during composting of medicinal herbal residues [60].

On the one hand, the research results provide a theoretical basis for the development of representative genera as compost decomposing agent. On the other hand, by adding corresponding representative genera in the corresponding composting phase, it will provides technical support for accelerating compost ripening process.

### 3.4 Correlation between microbial community and environmental factor

The correlation analysis showed that there was significant correlation among fungi and bacteria as well as between fungi and bacteria (shown in Table S1). There was a positive correlation between the fugal genera, *Chaetomium* and *Acrophialophora* (1.00). *Streptomyces* was significantly positive correlated with the bacterial genus *Olivibacter* (0.90) and *Bacillus* (0.85). The bacterial genus *Streptomyces* was significantly positive correlated with two fungal genera, *Chaetomium* (0.96) and *Acrophialophora* (0.97). The bacterial genus *Nonomuraea* was significantly positive correlated with the fungal genera *Thermomyces* (0.86). In addition, a significant negative correlation was also observed. For example, the representative genera of the maturation phase, *Pseudoxanthomonas*, was negatively correlated with three representative genera of the thermophilic phase, *Streptomyces* (–0.67), *Olivibacter* (–0.70), and *Thermomyces* (–0.79).

The representative genera of the maturation phase, *Thermomyces*, was negatively correlated with three representative genera of the thermophilic phase, *Streptomyces* (–0.62), *Olivibacter* (–0.70), and *Thermomyces* (–0.79).

Microbial positive or negative correlation reflects the symbiotic and competitive relationship of microorganisms in composting process, resulting in the succession of microbial niche and microbial community. There was a significant negative correlation between the fungal genus, *Myceliophthora* and the bacterial genus, *Brachybacterium* (–0.883). The negative correlation among microbial genera is determined by their different niches. For example, the abundance of *Brachybacterium* (1.3%) was significantly higher than that of *Myceliophthora* (0.1%) in the initial phase, while the abundance of *Myceliophthora* (42%) was significantly higher than that of *Brachybacterium* (0) in the maturation phase.

The representative genera of lignin degrading fungi include *Aspergillus*, *Chaetomium*, *Penicillium*, etc. [47]. Studies have shown that thermophilic actinomycetes such as *Nocardia*, *Streptomyces, and Thermoactinomyces* are the dominant bacteria for decomposing lignocellulose during high temperature composting [61, 62]. Lignin and its degradation products are important precursors of humus formation during composting. The effect of single bacteria, the effect of single fungi or single actinomycetes on the humification of compost is less than the synergistic effect of multiple microbial groups. The degradation of lignin by bacteria occurs in the primary metabolism stage, which mainly modifies the structure of lignin to a certain extent and rarely mineralizes lignin [63]. It has been reported that the degradation of lignin by *Streptomyces* and other *Actinomycetes* is in the primary metabolism stage. The degradation of lignin by fungi occurs in the secondary metabolism stage, and the lignin is completely mineralized to CO_2_ [44]. The correlation analysis showed that non‐fibrinous bacterial general, *Flavobacterium* and *Pseudomonas* were significant positive correlation with fungal genus *Aspergillus*, and a significant negative correlation with fungal genus *Myceliophthora*. It has been reported that non‐filamentous bacteria such as *Flavobacterium* and *Pseudomonas* can only degrade low molecular weight lignin and lignin degradation products [64]. This shows that bacteria, actinomycetes and fungi play a synergistic role in lignin degradation. The positive correlation between bacteria and fungi indicates that the humification process of GW compost is the result of the combined action of bacteria, actinomycetes, and fungi.

#### 3.4.1 Microbial metabolism functions of compost

**FIGURE 6:**
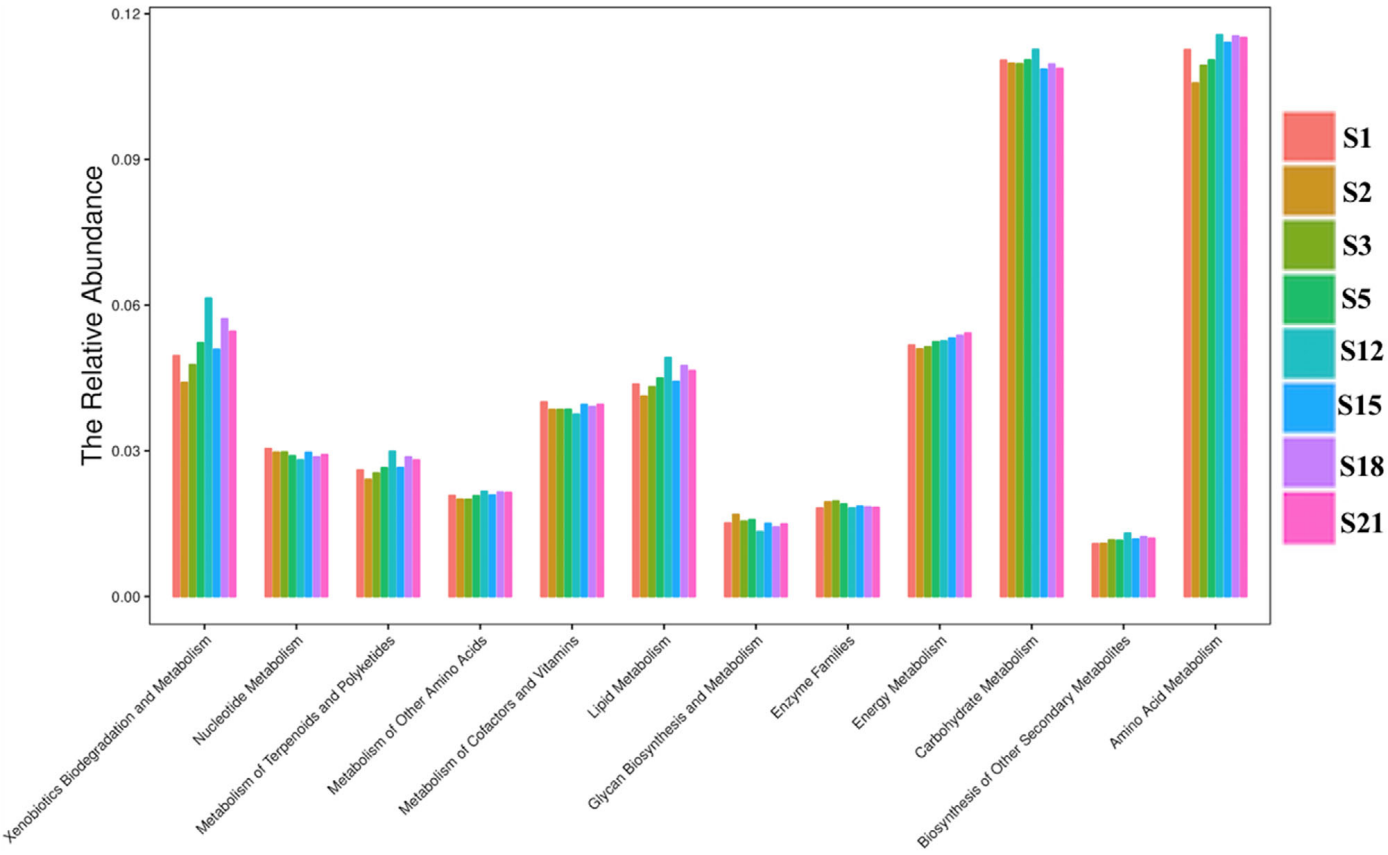
Analysis of metabolic pathway in GW composting process analyzed by KEGG level 2

The analysis of KEGG metabolism showed that the top four types of metabolism were amino acid metabolism, carbohydrate metabolism, energy metabolism, xenobiotics biodegradation and metabolism and lipid metabolism (Figure 6). These four types of metabolism are closely related to microbial mineralization and humification. Amino acid metabolism, carbohydrate metabolism and energy metabolism always play an important role in mineralization and humification. In the mineralization stage, microorganisms decompose organic carbon through carbohydrate metabolism and energy metabolism and assimilate 0inorganic nitrogen into amino acids and organic nitrogen. In the humification stage, lignocellulose is also slowly degraded accompanied by carbohydrate metabolism and energy metabolism. Amino acids can be used as raw materials for HA synthesis and have direct promotion on humus synthesis [22, 29]. The study showed that when the C/N ratio increased to a certain extent, the organic nitrogen oxidation process weakened, and the microbial organic nitrogen synthesis was significant [28]. With the decrease of temperature, the humification increased, and organic nitrogen was transformed into HA containing aromatic compounds [14].

Carbohydrate metabolism during composting can produce various compounds through the degradation of hemicellulose and cellulose [65]. In the mineralization stage of composting process, microorganisms preferentially decompose easily degradable substances such as sugar, starch, protein, lipid, and so on. With the continuous degradation of easily degradable substances, microorganisms obtain energy and assimilate organic nitrogen, which corresponds to carbohydrate metabolism, amino acid metabolism, lipid metabolism, and energy metabolism. In this study KEGG metagenome at Level 3 analysis showed that transporters had the highest abundance, followed by ABC transporters. This indicates that microorganisms exchange substances with the external environment through membrane transporters and ABC transporters. For example, the microorganisms secrete lignin degrading enzymes to the outside of cells and transports small molecules such as glucose into the inside of cells. The microorganisms degrade the refractory substances such as lignin and phenol during the humification stage [66]. In the GW composting process, in addition to a small amount of polymers and pesticides, the exogenous substances should be mainly the degradation products of lignin, polycyclic aromatic hydrocarbons, which corresponds to xenobiotics biodegradation and metabolism.

## 4 CONCLUDING REMARKS

The temperature, microbial genera, substrate, and product are different in four phases of composting process. In the calefactive phase of GW composting, various microorganisms use simple organic matter to rapidly reproduce, which provides conditions for incubating the thermophilic microorganisms and the arrival of the thermophilic phase. In the thermophilic phase, the thermophilic microorganisms decompose lignocellulose to produce FA and lignin core. In the cooling phase a large number of  humification microorganisms are screened out for the maturation phase. In the maturation period, microorganisms use FA, amino acids and lignin core as precursors to synthesize HA. HA and FA can regulate the composting process and lignocellulose degradation in the composting process. Bacteria, actinomycetes and fungi have synergistic effect on the degradation of lignocellulose. One bacterial genera, *Streptomyces*, and two fungal genera, *Myceliophthora* and *Aspergillus*, maintained high abundance in all phases of the compost.

## CONFLICT OF INTEREST

The authors declared that they have no conflicts of interest to this work. We declare that we do not have any commercial or associative interest that represents a conflict of interest in connection with the work submitted.

## Supporting information

Supplementary Table Correlation analysis of dominant phyla of microorganisms in GW composting process

## Data Availability

The data that support the findings of this study are available from the corresponding author upon reasonable request.
