# Supplementary material for: Study on dynamic changes of microbial community and lignocellulose transformation mechanism during green waste composting
Source: Eng Life Sci. 2022 Feb 5;22(5):376–90. doi: 10.1002/elsc.202100102 (PMC9077819; doi:10.1002/elsc.202100102)
Supplement: Supplementary file 1 — Supplementary Table Correlation analysis of dominant phyla of microorganisms in GW composting process [file ELSC-22-376-s001.docx]

Supplementary Table Correlation analysis of dominant phyla of microorganisms in GW composting process

|  | Streptomyces | Olivibacter | Enterobacter | Pseudonocardia | Microvirga | Bacillus | Nonomuraea | Pseudoxanthomonas | Actinomadura | Thermomonospora | Micromonospora | Myceliophthora | Aspergillus | Chaetomium | Penicillium | Acrophialophora | Thermomyces |
| --- | --- | --- | --- | --- | --- | --- | --- | --- | --- | --- | --- | --- | --- | --- | --- | --- | --- |
| Streptomyces | 1 |  |  |  |  |  |  |  |  |  |  |  |  |  |  |  |  |
| Olivibacter | 0.90 | 1 |  |  |  |  |  |  |  |  |  |  |  |  |  |  |  |
| Enterobacter | 0.74 | 0.83 | 1 |  |  |  |  |  |  |  |  |  |  |  |  |  |  |
| Pseudonocardia | -0.47 | -0.38 | -0.51 | 1 |  |  |  |  |  |  |  |  |  |  |  |  |  |
| Microvirga | 0.46 | 0.44 | 0.72 | -0.44 | 1 |  |  |  |  |  |  |  |  |  |  |  |  |
| Bacillus | 0.85 | 0.67 | 0.35 | -0.5 | 0.17 | 1 |  |  |  |  |  |  |  |  |  |  |  |
| Nonomuraea | -0.46 | -0.58 | -0.6 | 0.81 | -0.21 | -0.39 | 1 |  |  |  |  |  |  |  |  |  |  |
| Pseudoxanthomonas | -0.67 | -0.7 | -0.79 | 0.36 | -0.3 | -0.4 | 0.43 | 1 |  |  |  |  |  |  |  |  |  |
| Actinomadura | -0.34 | -0.21 | -0.42 | 0.95 | -0.3 | -0.36 | 0.76 | 0.33 | 1 |  |  |  |  |  |  |  |  |
| Thermomonospora | -0.21 | -0.03 | -0.38 | 0.51 | -0.42 | -0.08 | 0.11 | 0.54 | 0.59 | 1 |  |  |  |  |  |  |  |
| Micromonospora | 0.69 | 0.65 | 0.33 | -0.49 | -0.01 | 0.87 | -0.55 | -0.48 | -0.34 | 0.03 | 1 |  |  |  |  |  |  |
| Myceliophthora | 0.21 | 0.29 | 0.04 | 0.47 | 0.29 | 0.1 | 0.39 | 0.27 | 0.65 | 0.52 | -0.11 | 1 |  |  |  |  |  |
| Aspergillus | -0.13 | -0.09 | 0.11 | 0.02 | -0.41 | -0.29 | -0.25 | -0.36 | -0.21 | -0.15 | -0.14 | -0.64 | 1 |  |  |  |  |
| Chaetomium | 0.96 | 0.86 | 0.76 | -0.59 | 0.62 | 0.85 | -0.48 | -0.63 | -0.42 | -0.29 | 0.7 | 0.19 | -0.29 | 1 |  |  |  |
| Penicillium | 0.69 | 0.47 | 0.26 | -0.6 | -0.06 | 0.89 | -0.48 | -0.49 | -0.57 | -0.29 | 0.86 | -0.35 | 0.07 | 0.67 | 1 |  |  |
| Acrophialophora | 0.97 | 0.9 | 0.79 | -0.55 | 0.62 | 0.82 | -0.49 | -0.65 | -0.38 | -0.26 | 0.68 | 0.24 | -0.27 | 1 | 0.63 | 1 |  |
| Thermomyces | -0.62 | -0.7 | -0.79 | 0.77 | -0.35 | -0.42 | 0.86 | 0.75 | 0.73 | 0.48 | -0.5 | 0.37 | -0.25 | -0.63 | -0.51 | -0.64 | 1 |
